# Hippocampal volumetry to determine the resection side in patients with intractable non-lesional bilateral temporal lobe epilepsy

**DOI:** 10.1038/s41598-023-30151-8

**Published:** 2023-02-23

**Authors:** Go Seyama, Koji Iida, Kota Kagawa, Masaya Katagiri, Akitake Okamura, Hiromi Morioka, Nobutaka Horie

**Affiliations:** 1 Department of Neurosurgery, Graduate School of Biomedical and Health Science, Hiroshima University, 1-2-3 Kasumi, Minami-ku, Hiroshima, 734-8551 Japan; 2 Department of Neurosurgery, Takanobashi Central Hospital, 2-4-16 Kokutaiji-chou, Naka-ku, Hiroshima, 730-0042 Japan; 3 Department of Neurosurgery, Mazda Hospital, 2-15 Aosakiminami, Futyuu-chou, Aki-gun, Hiroshima, 735-8585 Japan; 4 Epilepsy Center, Hiroshima University Hospital, 1-2-3 Kasumi, Minami-ku, Hiroshima, 734-8551 Japan

**Keywords:** Neuroscience, Neurology

## Abstract

Bilateral Temporal lobe epilepsy (BTLE) cases may result in poor surgical outcomes due to the difficulty in determining/localizing the epileptogenic zone. In this study, we investigated whether hippocampal volume (HV) would be useful for the determination of the best resection side in BTLE. Eighteen cases of BTLE determined by a scalp video electroencephalogram (SVEEG) underwent resection via intracranial electroencephalography (IVEEG). Patients with lesions or semiologically determined focus lateralization were excluded. In addition to SVEEG, an epilepsy protocol magnetic resonance imaging (MRI) including hippocampus fluid-attenuated inversion recovery (FLAIR) and HV, 18F-fluorodeoxyglucose positron emission tomography (FDG-PET), single-photon emission computed tomography with 123I-iomazenil (IMZ-SPECT), and magnetoencephalography (MEG) were performed for the preoperative evaluation of the lateralization. The resection side was determined based on the IVEEG results, and the seizure outcome at two years postoperatively was classified as either a well-controlled seizure outcome (Engel class I), or residual (classes II–V). We used a Fisher's exact test to compare the concordance between the determination of the epileptic focus by each modality and the resected side where patients achieved a well-controlled seizure outcome. Seizures were well controlled in 9/18 patients after surgery. Eight out of 11 patients (72.7%), in whom the HV results (strongly atrophic side) and the resection side were matched, had well-controlled seizure outcomes (P = 0.0498). The concordance of other presurgical evaluations with the resection side was not significantly related to a well-controlled seizure outcome. HV may be a useful method to determine the optimal resection side of the epileptic focus/foci in cases of suspected BTLE.

## Introduction

Temporal lobe epilepsy (TLE) is a disease in which surgery is expected to significantly improve seizures compared to drug therapy^1^. It has been reported that seizures resolved in 55–70% of patients within 1 year after surgery^2^. Since the elimination of seizures is expected to improve the quality of life for epilepsy patients, surgery tends to be considered for further seizure control in TLE.

However, in epilepsy patients with suspected TLE, a scalp video electroencephalography (SVEEG) will occasionally indicate seizures independently occurring in the temporal lobes bilaterally. Such TLE is called bilateral temporal lobe epilepsy (BTLE), whereas TLE in which seizures begin in only one temporal lobe is called unilateral temporal lobe epilepsy (UTLE)^3^.

In BTLE, seizures occur independently or simultaneously in both temporal regions on SVEEG. Therefore, it is difficult to determine the resection side on EEG findings. Although single-photon emission computed tomography with 123I-iomazenil (IMZ-SPECT) and fluorodeoxyglucose positron emission tomography (FDG-PET) can be used to determine the resection side in BTLE as well as in UTLE, they have not been reported to be particularly useful for BTLE. Therefore, invasive intracranial video electroencephalography (IVEEG) is often performed in cases where BTLE is suspected^4,5^. Alternatively, there are reports that some BTLE patients may not be the best candidates for surgery because even IVEEG is not sufficient to determine the optimal resection side^6^.

In the present study, we focused on the volume of the hippocampus for this determination. In previous reports of UTLE, hippocampal atrophy was useful in determining the resection side, but there is no similar study for BTLE^7^. We used the difference in hippocampal volumes (HV) between the right and left sides of the brain in non-pathological BTLE patients. We examined the relationship between the left and right values from several modalities, including hippocampal volume, and compared them to favorable postoperative seizure outcomes in non-pathological BTLE patients.

## Methods

### Patient selection

We retrospectively reviewed the clinical data of patients who underwent surgical treatment for intractable epilepsy at the Department of Neurosurgery Epilepsy, Hiroshima University Hospital Epilepsy between 2006 and 2018.

Our criteria for BTLE were as follows: (1) ictal EEG findings indicated that the bilateral temporal regions were involved simultaneously, or (2) alternately began from both left and right temporal regions.

All pre-operative findings were discussed at a multi-disciplinary epilepsy conference. Pre-operative evaluation included etiology, seizure semiology, SVEEG, MRI, hippocampal volume (HV), FDG-PET, IMZ-SPECT, MEG, and IVEEG.

Patients who met the following conditions were included. (1) Patients diagnosed as having BTLE according to our above criteria. (2) Patients who received bilateral intracranial electrode implantation to determine the epileptogenic zone, including a hippocampal depth electrode. (3) No consistent clinical sign to determine seizure lateralization. (4) No abnormal lesions based on MRI (e.g., tumor, encephalomalacia, and vascular abnormality). (5) At least 2 years of follow-up after resection surgery.

### Ethics statement

The present study was approved by the Ethics Committee for Epidemiology at Hiroshima University (E-749-1). Informed consent was obtained in the form of an opt-out on the website. Those who opted out were excluded. All methods were performed in accordance with the relevant guidelines and regulations.

### Preoperative evaluation and determination of resection side

#### Scalp video-EEG monitoring

We performed prolonged SVEEG using an international 10-20 Scalp-Electrode Placement System with a single-reference electrode (BMSI 4000 and 6000, Nicolet Biomedical Inc., Madison, Wisconsin, USA). We used bilateral sphenoidal electrodes or T1/T2 electrodes. The SVEEG was continued for 2–7 days to capture all types of habitual seizures. We analyzed interictal and ictal epileptiform discharges at a sampling rate of 500 Hz^8^.

#### Magnetic resonance imaging

All patients underwent MRI on both a 1.5T MRI scanner (Excelart Vantage, Toshiba Medical Systems Corporation, Otawara, Japan) at the Division of Radiology, Hiroshima-Chuo-Kenshin-Sho, and a 3.0T MR scanner (Signa EXITE HD 3T, GE Medical Systems, Waukesha, Wisconsin, USA) in the Division of Radiology, Hiroshima University Hospital.

Our diagnostic protocol for epilepsy consisted of T1-weighted and T2-weighted imaging in three orthogonal planes, axial fluid attenuated inversion recovery (FLAIR) imaging, and thin coronal (3 mm) and axial (3 mm) T2-weighted and FLAIR images, which were parallel to and perpendicular to the long axis of the hippocampus. T1-weighted images by three-dimensional real inversion recovery were also acquired to observe the entire shape of the hippocampus in the axial direction (1 mm)^9,10^.

Hippocampal sclerosis (HS) is a characteristic finding in TLE and is reported in approximately 56% of TLE cases^11^. MRI features of HS include reduced hippocampal volume and increased signal intensity on FLAIR imaging. In our study, many cases of BTLE included bilateral high signal intensity on FLAIR concurrent with atrophy. Therefore, we decided to evaluate hippocampal atrophy separately from the presence or absence of high signal intensity on FLAIR. For hippocampal atrophy, we focused on the difference between the left and right hippocampal volumes, as described below. For high signal intensity on FLAIR, the side with a more pronounced high signal was considered to indicate the localization of seizure foci.

In the cases with MR lesions, a higher rate of seizure-free outcomes can be expected in TLE with lesions than in TLE without apparent lesions^12^. In the sense that the resection side in such cases is predictable, BTLE may have a similarly high rate of seizure improvement. In this study, TLE cases with lesions were excluded because lesions were suggestive of the seizure onset side.

#### Volumetric analysis of the hippocampus

Hippocampal volumetry was performed on images acquired by a 1.5T scanner (Excelart Vantage, Toshiba Medical Systems Corporation, Otawara, Japan) in the Division of Radiology, Hiroshima-Chuo-Kenshin-Sho, using a protocol for HV as follows: coronal (1 mm) planes on T1-weighted images (three-dimensional real inversion recovery) parallel and perpendicular to the hippocampal long axis.

The anterior boundary of the hippocampus was manually traced on the first section where the amygdala could not be observed, and the posterior boundary was traced on the section before the crus of the fornix, by J.K., a radiology technologist at the Division of Radiology, Hiroshima-Chuo-Kenshin-Sho. The technologist was blinded to the other data. We used MRI volume calculation software for a personal computer (M-Power, version 2.3; Toshiba Medical Systems Corporation) to measure the HV. Iida et al. have used this method previously to report that normal HV in healthy individuals was 1.56 ± 0.23 cm^3^^10^.

To determine the resection side for TLE, we noted a difference in the size of the left and right hippocampus. The differences in the HV between the left and right sides or a comparison with the volume in healthy subjects are frequently used. In this study, the left and right sides were compared and the more atrophic side was determined to coincide with the resected side.

#### IMZ-SPECT

IMZ-SPECTs were performed during the interictal state with the central benzodiazepine antagonist 123I-iomazenil. IMZ-SPECT is a widely used tool to measure regional cerebral blood flow (rCBF) noninvasively, and a decrease in rCBF is considered to indicate the localization of a seizure foci^13^. The area of decreased rCBF was defined as the area of visually decreased RI accumulation, and its validation was determined through discussion at a preoperative multidisciplinary patient management conference (PMC).

#### FDG-PET scan

The FDG-PET brain scan was performed on a discovery ST Elite PET/CT scanner (GE Medical Systems, Milwaukee, WI, USA) at Hiroshima-Heiwa Clinic, to evaluate focal abnormalities in regional cerebral metabolic rate (rCMR). Low rCMR indicates areas of hypometabolism, suggestive of the epileptogenic foci^14^. The area of hypometabolism was visually confirmed and its validation was determined through discussion at a preoperative PMC.

#### MEG recording

MEG recordings were digitized at 600.615 Hz using the Neuromag System (a whole-head 306 type, Elekta-Neuromag O.Y., Helsinki, Finland). We simultaneously recorded EEG using 19 scalp electrodes, with an electrocardiogram and electro-oculograms. The MEG recording included three blocks of 10 min in an awake or spontaneously asleep state. We classified the distribution of equivalent current dipole (ECD) into clusters and scatters during the interictal phase^15^.

#### Intracranial video-EEG monitoring

Intracranial video-EEG monitoring was performed to localize the epileptogenic zone (BMSI 4000 and 6000, Nicolet Biomedical Inc., Madison, Wisconsin, USA). The EEG sampling rate was set at 1024 Hz for recording the details of the seizure propagation. Depth electrodes were placed in the hippocampus bilaterally in all patients. The IVEEG was continued for 3–15 days to capture all types of habitual seizures (average of 8 days). The majority of the seizure onset zones were identified visually on IVEEG traces during long-term IVEEG monitoring^15^. Lateralized seizure activity was seen in at least one seizure recording with IVEEG.

### Surgical procedure

The strategies for IVEEG implantation were discussed during a PMC and a review of the multiple modalities including 1.5 or 3T MRI, SVEEG, FDG-PET, IMZ-SPECT, and MEG was conducted. Based on the consensus hypothesis of the epileptogenic zone at the PMC, the IVEEG electrodes were implanted and IVEEG was performed. The location and extent of the proposed resection were discussed during a second PMC, after reviewing the results of the IVEEG and functional mapping.

For most of the cases, we performed a classical anterior temporal lobectomy (ATL) with amygdalo-hippocampectomy for TLE. The temporal lobe was resected less than 4.5 cm in length in the language-dominant side and less than 6 cm in the non-dominant side^1^. In principle, the hippocampus was resected, but in patients with preserved higher memory functions, the hippocampus was not resected and multiple subpial resections (MST) were performed. For patients with mesial TLE diagnosed by IVEEG, a selective amygdalo-hippocampectomy (SAH) was performed in the language-dominant hemisphere, and an ATL was performed in the language-non-dominant hemisphere. If an additional resection of the posterior temporal cortex was necessary, extended ATL was performed^10^.

### Seizure outcome

After the operation, patients were followed up for at least two years to observe surgical outcomes.

Seizure outcome was classified two years after the surgery into two groups: a well-controlled group (Engel class I), and a residual group (Engel class II–V), according to the system proposed by Engel^16^.

### Statistical analysis

We calculated sensitivity and specificity as a percentage from the tables as follows: Sensitivity = Total cases in well-controlled group/Cases with each modality determining resection side matched with correct resection side; Specificity = Total cases in residual group/Cases with each modality determining resection side not matched with correct resection side.

We used a Fisher's exact test to compare the concordance rate between the findings by each modality used to determine laterality and the actual resected side when patients achieved well-controlled seizure outcomes (Engel class I). Analyses were performed using JMP pro15. Differences were statistically significant if the p-value was < 0.05. It was judged that the diagnosis of the correct resection side by presurgical evaluation was achieved when the resection side of cases with well-controlled seizure outcomes matched the resection side as determined by the presurgical evaluation.

## Results

### Patient demographics

Between 2006 and 2018, 52 patients underwent IVEEG. Twenty-seven out of 52 patients were considered to have TLE, and 21 patients had bilateral electrodes implanted due to the presence of BTLE. Three patients who did not meet the inclusion criteria (one case with a lesion on MRI and two cases with only electrode removal because no determination of the resection side was obtained from the IVEEG results) were excluded. In total, 18 cases were included in the subsequent analyses.

Table 1 shows the patient demographics. Eleven of 18 patients were men. The mean age at the time of surgery was 37.0 ± 11.5 (mean ± standard deviation) years, ranging from 21 to 63 years. The mean duration from the onset of the seizure disorder to surgery was 22.6 ± 13.7 years. A classical ATL was performed in six patients (33.3%), a SAH was performed in five patients (27.8%), and an ATL with MST was performed in three patients (16.7%). Extended ATL was performed in four patients (22.2%). After surgery, the mean follow-up duration was 71.3 ± 33.0 months. The outcome did not significantly vary by gender, age at the time of surgery, or duration of the onset of epilepsy disorder to the time of surgery (Table 2). Six patients had a history of encephalitis or meningitis. Five out of six patient outcomes were classified as residual.

**Table 1:** Patients characteristics.

No./age (years)/sex	Epilepsy duration (years)	Type of seizure	Seizure frequency	Etiology	Surgery	Histopathological diagnosis
1/46/F	5	FIAS/FBTCS	4, 5/week	Normal	ATL + MST	No lesion
2/39/M	25	FIAS	1, 2/week	Normal	Extended ATL	No lesion
3/27/M	12	FIAS/FBTCS	2, 3/month	Normal	ATL	No lesion
4/50/F	36	FIAS	1, 2/month	Normal	ATL	HS
5/32/M	10	FIAS/FBTCS	1, 2/month	Normal	ATL	FCD type1
6/44/M	37	FIAS	1, 2/month	Meningitis	ATL	FCD type1
7/46/F	36	FIAS/FBTCS	1/month	Normal	ATL + MST	No lesion
8/22/M	5	FIAS/FBTCS	1/week	Normal	ATL + MST	No lesion
9/41/F	35	FIAS/FBTCS	4, 5/month	Normal	SAH	HS
10/21/M	16	FIAS/FBTCS	1, 2/month	Meningitis	SAH	No lesion
11/33/M	31	FIAS/FBTCS	4, 5/month	Normal	Extended ATL	No lesion
12/53/F	50	FIAS/FBTCS	2, 3/week	Normal	Extended ATL	No lesion
13/24/M	18	FIAS/FBTCS	10/week	Normal	Extended ATL	No lesion
14/24/F	4	FIAS	1, 2/week	Viral encephalitis	ATL	FCD type1
15/63/F	32	FIAS/FBTCS	1, 2/month	Normal	SAH	No lesion
16/37/M	28	FIAS/FBTCS	1, 2/month	Meningoencephalitis	SAH	No lesion
17/35/M	3	FIAS/FBTCS	2, 3/month	Encephalitis	SAH	HS
18/29/M	24	FIAS	1, 2/week	Herpes encephalitis	ATL	HS

F, female; M, male; Rt., right; Lt., left; Bil, bilateral; FIAS, focal Impaired awareness seizure; FBTCS, focal to bilateral tonic-chronic seizure; ATL, anterior temporal lobbectomy with amygdalohippocampectomy; ATL + MST, anterior temporal lobectomy with multiple subpial resections; SAH, selective amygdalo-hippocampectomy; HS, Hippocampal sclerosis; FCD; Focal cortical dysplasia

**Table 2:** Patients characteristics and clinical outcomes.

Characteristic	Engel I	Engel II–V	Overall	p-value
Male sex (%)	55.6	66.7	61.1	0.63
Age at surgery (years)	40.9 ± 13.5	33.1 ± 8.9	37.0 ± 11.8	0.17
Age at seizure onset (years)	13.4 ± 9.0	15.4 ± 13.3	14.4 ± 11.1	0.71
Epilepsy duration (years)	27.5 ± 14.4	17.7 ± 12.6	22.6 ± 14.1	0.14

Plus-minus values are mean ± SD. Epilepsy duration was the duration from the onset of seizures to epilepsy surgery. Between the well-controlled and residual groups, sex was compared using the chi-square test, and other were compared using the student's t-test.

### Surgical outcome

The determination of laterality by each modality is listed in Table 3. In each case, the information provided by each modality to determine the best side to resect, as well as the actual side resected, and the 2-year postoperative outcome are described. Nine patients (50%) achieved well-controlled seizure outcomes within Engel class I at a 2-year follow-up. There were no surgical complications.

**Table 3:** Resection side diagnosis at each evaluation and surgical outcome.

No./age (years)/sex	HV (cm^3^)			MRI FLAIR image	SVEEG		FDG-PET	IMZ-SPECT	MEG	IVEEG		Resection side	Outcome (Engle score)
	Smaller side	L	R		Ictal	Interictal				Ictal	Interictal		
1/46/F	L	1.9	2.39	Normal	L(0), R(5), bi(1)	R = L	No data	L	R	0	R > L	R	IIa
2/39/M	R	2.05	1.04	R	L(0), R(4), bi(4)	R > L	R	No data	R	L(0), R(9), bi(0)	R > L	R	Ib
3/27/M	L	0.9	1.05	bil (L > R)	L(2), R(1), bi(1)	L > R	bil	L	L	L(0), R(36), bi(0)	R > L	R	IIc
4/50/F	R	0.89	0.79	bil (R > L)	L(1), R(5), bi(0)	L > R	R	L	No spike	L(0), R(16), bi(0)	L > R	R	Ia
5/32/M	L	1.08	1.2	Normal	L(4), R(0), bi(1)	L > R	L	L	L	L(1), R(0), bi(0)	L > R	L	Ia
6/44/M	R	1.28	0.84	R	L(0), R(1), bi(6)	R > L	R	L	No spike	L(1), R(5),bi(0)	R > L	R	Ia
7/46/F	R	1.38	1.25	Normal	L(2), R(4), bi(3)	R > L	Normal	L	No spike	L(6),R(0),bi(0)	R > L	L	IIc
8/22/M	L	1.04	1.3	Normal	L(2), R(0), bi(5)	L > R	R	R	R	L(0), R(2), bi(0)	L > R	R	Ia
9/41/F	L	0.68	1.45	L	L(0), R(0), bi(4)	L > R	L	No data	No spike	L(6), R(0), bi(0)	L > R	L	Ia
10/21/M	L	1.39	1.46	L	L(2), R(1), bi(2)	L > R	R	L	No spike	L(5), R(10), bi(0)	L > R	L	IV
11/33/M	L	1.24	1.36	Normal	L(0), R(2), bi(1)	R > L	R	R	R	L(0), R(1), bi(0)	R > L	R	IIa
12/53/F	L	0.5	1.42	L	L(3), R(0), bi(0)	L > R	L	L	No data	L(2), R(0), bi(0)	L > R	L	Ia
13/24/M	R	1.24	1.07	Normal	L(0), R(0), bi(4)	R > L	L	L	R	L(0), R(3), bi(2)	R > L	R	Ib
14/24/F	L	1.09	1.17	Normal	L(3), R(7), bi(0)	R > L	R	L	No spike	L(3), R(4), bi(0)	L > R	R	IV
15/63/F	R	1.27	1.23	bil (R > L)	L(0), R(2), bi(2)	L > R	R	R	R	L(1), R(1), bi(0)	L = R	R	Ia
16/37/M	L	1.15	1.22	normal	L(1), R(0), bi(1)	L > R	L	R	R	L(2), R(2), bi(0)	R > L	R	V
17/35/M	L	0.64	0.77	bil (L > R)	L(1), R(12), bi(0)	L > R	L	L	L	L(4), R(3), bi(0)	L > R	L	V
18/29/M	R	1.54	1.16	bil (R > L)	L(3), R(5), bi(0)	L = R	R	R	L	L(3), R(3), bi(2)	R > L	R	IIIb

F, female; M, male; L, left; R, right; bil, bilateral; HV, hippocampal volume; SVEEG, scalp video electroencephalograph; FDG-PET, fluorodeoxyglucose-position emission tomography; IMZ-SPECT, iomazenil single photon emission computed tomography; MEG, magnetoencephalography; IVEEG, intracranial video electroencephalograph.

### Analysis of data

Table 4 shows the concordance rate between the findings by each modality and the resected side where patients achieved well-controlled seizure outcomes. The determination of the best side for resection based on HV was significantly concordant with the actual resected side where patients achieved well-controlled seizure outcomes (p = 0.0498). No significant agreement was observed with the other preoperative evaluations.

**Table 4:** Analysis of data.

		N	Seizure		Sensitivity		Specificity		Fisher p-value
		y/n	Engel I	Engel II–V					
Hippocampal volume		11/7	8/1	3/6	8/11	(72.7%)	6/7	(85.7%)	0.0498*
MRI FLAIR image		9/9	6/3	3/6	6/9	(66.7%)	6/9	(66.7%)	0.3469
SVEEG	Ictal side	11/7	6/3	5/4	6/11	(54.5%)	4/7	(57.1%)	1.0000
	Interictal side	10/8	6/3	4/5	6/10	(60.0%)	5/8	(62.5%)	0.6372
FDG-PET		12/5	8/1	4/4	8/12	(66.7%)	4/5	(80.0%)	0.1312
IMZ-SPECT		10/6	4/3	6/3	4/10	(40.0%)	3/6	(50.0%)	1.0000
MEG		9/8	5/3	4/5	5/9	(55.6%)	5/8	(62.5%)	0.6372
IVEEG	Ictal side	15/3	8/1	7/2	8/15	(53.3%)	2/3	(66.7%)	1.0000
	Interictal side	13/5	6/3	7/2	6/13	(46.2%)	2/5	(40.0%)	1.0000

SVEEG, scalp video electroencephalogram; FDG-PET, fluorodeoxyglucose-position emission tomography; IMZ-SPECT, iomazenil single photon emission computed tomography; MEG, magnetoencephalography; IVEEG, intracranial video electroencephalograph.

#### SVEEG findings

All 18 patients underwent SVEEG. Bilateral abnormalities were found in all cases of interictal EEG as well as ictal SVEEG. The ictal SVEEG sensitivity and specificity were 6/11 (54.5%) and 4/7 (57.1%). The interictal SVEEG sensitivity and specificity were 6/10 (60%) and 4/8 (62.5%), respectively.

#### MRI findings

All 18 patients had their MRIs reviewed and seven patients had a normal MRI. Five patients had bilateral high signal intensity in the mesial temporal lobe on FLAIR imaging, with a sensitivity and specificity of 6/9 (66.7%) and 6/9 (66.7%).

#### Hippocampal volumetry findings

In all 18 cases, HV was performed. When comparing the left and right hippocampi, 11 cases were smaller on the left side, and 7 cases were smaller on the right side. No cases had the same hippocampal volume bilaterally. The sensitivity of the HV atrophic side was 8/11 (72.7%) and the specificity was 6/7 (85.7%). Compared to the normal HV reported previously by our group, 88.9% cases of suspected BTLE had bilateral hippocampal atrophy^10^.

The excised tissues were submitted for histopathological analysis, and hippocampal sclerosis was confirmed in 22.2% of the cases (Table 1). Of the 4 cases with hippocampal sclerosis, 2 cases were classified during follow up as well-controlled, one was class IIIb, and the other was class V.

#### IMZ-SPECT/FDG-PET findings

IMZ-SPECT and FDG-PET were performed on 16 and 17 patients, respectively. The sensitivity and specificity of IMZ-SPECT were 4/10 (40%) and 3/6 (50%), respectively. The sensitivity and specificity of FDG-PET were 8/12 (66.7%) and 80.0% (4/5), respectively. Both values were lower than those from the HV analysis.

#### MEG findings

MEG was performed in 17 of 18 patients. In six cases, there was no spike, and the dipole could not be distinguished. The sensitivity was 5/9 (55.6%) and the specificity was 5/8 (62.5%).

#### IVEEG findings

The sensitivity of ictal IVEEG was 8/15 (53.3%). In 10 cases with SVEEG-suspected BTLE, the IVEEG indicated a unilateral seizure onset. In five cases, the seizure onset was bilateral, but biased to one side, and in three cases, seizure onset was equal on both sides.

The seizures resolved in 70% of the patients with unilateral seizure onset indicated by IVEEG, and in 25% of the patients with bilateral seizure onset.

On the interictal EEG, all patients had bilateral abnormal epileptic waves, with a sensitivity of 6/13 (46.2%) and a specificity of 2/5 (40%).

## Discussion

In this study, we investigated which non-invasive variable could predict the best side for surgical resection that resulted in well-controlled seizure outcomes in BTLE. Although neither sensitivity nor specificity in BTLE was particularly high for any of the tests, a significant agreement was indicated only for HV. The present study showed that HV may be the most useful measure to predict the best resection side for well-controlled seizure outcomes among these non-invasive studies in BTLE.

### Preoperative examination using multimodal evaluation

FDG-PET, IMZ-SPECT, and MEG have been reported to be effective tests to identify the side of epileptic focus in UTLE. However, in the present study, none of these tests could significantly predict the best side for resection to result in a well-controlled outcome in BTLE.

This finding may be due to differences in the underlying physiological processes assessed by each modality. The preoperative evaluations performed in this study can be divided into three main categories. The first includes nuclear medicine scans, FDG-PET, and IMZ-SPECT, which assess metabolism and blood flow alterations. The second is neurophysiological examinations, such as EEG and MEG. Lastly, there are diagnostic radiological examinations, such as HV and hippocampal FLAIR high signal intensity.

The nuclear medicine scans indicate areas of hypometabolism and decreased regional cerebral blood flow (rCBF), which more accurately reflects the degree of cerebral dysfunction. Areas of cerebral dysfunction may overlap with areas of the epileptogenic zone. This can be useful if the dysfunction only encompasses a limited region. However, the epileptogenic region may not be accurately delineated in cases with large areas of dysfunction, multiple areas of dysfunction, or temporary loss of function^17^. For these reasons, it is expected to be difficult to determine the side of resection in the case of BTLE as well.

The lack of significant differences in interictal EEG and magnetoencephalogram may be because the results of both tests depend on the test’s timing. Since the number of ictal and interictal spikes is dependent on the test duration and timing, a short test time may not allow for an accurate evaluation. The same is true for magnetoencephalography. In fact, in this study, results could not be obtained in 7/18 cases due to a failure to record spikes during the MEG test. For tests whose results depend on the timing of the test, it may be necessary to reevaluate the test duration.

Finally, the diagnostic radiological examinations that exhibited significant differences in this study are not affected by the time or timing of the examination and can represent the patient's state at the time the examination was performed. In particular, atrophy is an irreversible change, and once atrophied, sites cannot be restored. It was speculated that this result may be the reason why a significant difference was obtained in the radiological diagnosis while the other tests did not show substantial differences.

### Pathogenesis of BTLE

There are various theories about the pathogenesis of BTLE, which can be divided into two main categories. First, there are patients who have bilateral or multiple seizure onset from the beginning, and second, there are patients who have a unilateral seizure onset, which progresses to bilateral over time. For example, encephalitis may cause seizures that are bilateral from the onset of the disorder. In such cases, it may be difficult to obtain well-controlled seizure outcomes by a unilateral surgical resection^18^. However, even with BTLE, there are some cases in which the seizure disorder resolves after a unilateral surgery^5^. Therefore, not all cases are bilateral in nature, but many cases do progress to have bilateral foci.

The theory of secondary epileptogenesis may be useful as a mechanism for the development of a contralateral focus in patients with TLE. This concept indicates that the neural connectivity between an actively discharging epileptogenic site and other sites gradually induces similar seizure-like behavior in a normal network^19,20^. Because the network is created gradually over time, a test that can show changes over time may be useful for the resection diagnosis of BTLE.

### Usefulness of HV in BTLE

It is known that hippocampal atrophy occurs in TLE. Hippocampal atrophy has also been reported as useful to determine the laterality of the seizure focus. This is because hippocampal atrophy is the result of brain tissue damage from frequent seizures^21,22^. The fact that hippocampal atrophy is not present at the onset of TLE, but is evident several years later, suggests a development to hippocampal atrophy from underlying pathophysiology^23^. It has also been reported that brain atrophy is not limited to the ipsilateral hippocampus, but extends into adjacent lobes, as well as the contralateral hippocampus^24,25^. This may be due to the complex nature of the connectivity from the hippocampus. In particular, the ventral hippocampal commissural system has been reported to be bilaterally connected^26^ and is considered to be closely related in anatomical origin. It is predicted that this strong bilateral hippocampal connectivity causes seizures to affect the non-ablative side of the hippocampus, causing brain damage.

We also examined the relationship between the duration of the TLE disorder and hippocampal atrophy. Jokeit et al.^27^ reported a bilateral decline in hippocampal volume, glucose metabolism, and Wada hemispheric memory performance with increasing duration of refractory TLE. This finding is because seizures cause progressive damage to the hippocampus over time.

Iida and colleagues examined the hippocampal volume of the non-resected side over time in TLE patients in whom seizures resolved after surgery. They reported that atrophy of the contralateral hippocampus progressed even after surgery^10^. This result indicates that once the hippocampus is damaged, atrophy progresses over time even after the seizures have been controlled. A report examining the left and right HV after resection in patients with TLE found that the hippocampus contralateral to the lobectomy was significantly smaller than the hippocampus of controls but significantly larger than the hippocampus ipsilateral to the lobectomy^28^. In the UTLE, the hippocampus on the resected side was more atrophic than the hippocampus on the unresected side, which is expected because it has been subjected to damage over a longer period. The hippocampus on the non-resected side, which was not the initial seizure focus, may have been affected by the dense intracranial connectivity and an atrophic process, which began after that of the originating side. In other words, the more atrophied hippocampal side may be the original seizure origin.

In this study, we compared left–right differences in hippocampal volume with MRI data. Hippocampal atrophy was diagnosed visually, but if differences are subtle or if the atrophy is bilateral, it could be difficult to determine. By the logic of secondary epileptogenesis, HV may be the only preoperative test in which the measurable changes correlate with time since the onset of the seizure disorder. The significant difference found in HV may be due to the ability to capture changes in volume due to damage from seizures, and that cannot be measured by FDG-PET or IMZ-SPECT, which assess the region of functional decline, or by EEG or MEG, in which the results can change depending on the timing of the test.

### Limitations

If there is a history of encephalitis or meningitis, a seizure disorder is more likely to be multifocal. If the brain is affected by multiple foci simultaneously, the degree of progression in hippocampal atrophy may not be effective for determining the side of resection. Even in this study, the postoperative seizure resolution rate was low in patients with pre-existing encephalitis or meningitis, which may have hampered the determination of the resection side by HV. To obtain more accurate results, it is necessary to develop an automated method to empirically determine hippocampal atrophy, hypoaccumulation, and hypometabolic regions (rather than visually examine them) to improve reproducibility. Additionally, it will be important to replicate the current study using a larger sample size.

## Conclusion

Among the preoperative evaluation methods to determine the best side for resection in BTLE, only the evaluation of HV atrophy was significantly concurrent with postoperative seizure outcome. Although hippocampal atrophy has been reported to be useful to determine the side of resection in UTLE, it was also proven to be useful in BTLE.

In this study, we were able to evaluate the side of hippocampal atrophy more accurately by quantifying the difference between the left and right sides. By examining the side of the hippocampus that is atrophic, it may be possible to determine the optimal resection side in BTLE.

## Supplementary Information

Supplementary Information.

## Data Availability

All data generated or analyzed during this study are included in this published article (and its supplementary information files).
